# Neuroprotective Potential of Dendritic Cells and Sirtuins in Multiple Sclerosis

**DOI:** 10.3390/ijms23084352

**Published:** 2022-04-14

**Authors:** Francesco Piacente, Marta Bottero, Andrea Benzi, Tiziana Vigo, Antonio Uccelli, Santina Bruzzone, Giovanni Ferrara

**Affiliations:** 1 Department of Experimental Medicine (DIMES), University of Genova, Viale Benedetto XV, 1, 16132 Genoa, Italy; francesco.piacente@unige.it (F.P.); andreeabenzi@gmail.com (A.B.); 2 IRCCS Ospedale Policlinico San Martino, Largo Rosanna Benzi 10, 16132 Genova, Italy; marta.bottero@hsanmartino.it (M.B.); tiziana.vigo@hsanmartino.it (T.V.); auccelli@neurologia.unige.it (A.U.); giovanni.ferrara@hsanmartino.it (G.F.)

**Keywords:** dendritic cells, multiple sclerosis, sirtuins, neuroinflammation, neuroprotection, immunomodulation

## Abstract

Myeloid cells, including parenchymal microglia, perivascular and meningeal macrophages, and dendritic cells (DCs), are present in the central nervous system (CNS) and establish an intricate relationship with other cells, playing a crucial role both in health and in neurological diseases. In this context, DCs are critical to orchestrating the immune response linking the innate and adaptive immune systems. Under steady-state conditions, DCs patrol the CNS, sampling their local environment and acting as sentinels. During neuroinflammation, the resulting activation of DCs is a critical step that drives the inflammatory response or the resolution of inflammation with the participation of different cell types of the immune system (macrophages, mast cells, T and B lymphocytes), resident cells of the CNS and soluble factors. Although the importance of DCs is clearly recognized, their exact function in CNS disease is still debated. In this review, we will discuss modern concepts of DC biology in steady-state and during autoimmune neuroinflammation. Here, we will also address some key aspects involving DCs in CNS patrolling, highlighting the neuroprotective nature of DCs and emphasizing their therapeutic potential for the treatment of neurological conditions. Recently, inhibition of the NAD^+^-dependent deac(et)ylase sirtuin 6 was demonstrated to delay the onset of experimental autoimmune encephalomyelitis, by dampening DC trafficking towards inflamed LNs. Thus, a special focus will be dedicated to sirtuins’ role in DCs functions.

## 1. Introduction

Inflammation is a physiological process concerning the vascularized connective tissue and the immune system that can be triggered by a variety of factors, including pathogens, genetic predisposition, apoptotic cells, and toxic compounds. These factors may induce inflammatory responses in all the tissues, potentially leading to the development of inflammatory diseases. Inflammation can be divided into three types based on the timing of the process in response to the injurious event: acute, which occurs immediately after injury and lasts for a few days [1]; chronic, which may last for months or even years when acute inflammation fails to resolve the problem [2]; subacute, which is a transformational period from acute to chronic, lasting less than 8 weeks [1,3]. Usually, during acute inflammatory responses, cellular and molecular events and interactions efficiently minimize impending injury or infection. However, uncontrolled acute and subacute inflammation may become chronic, contributing to a variety of chronic inflammatory diseases [4]. Recently, it has become evident that the resolution of inflammation is a biosynthetically actively driven process, precisely regulated and controlled by specialized pro-resolving mediators. Coordinated resolution programs initiate shortly after inflammatory responses begin. These fine biological mechanisms drive the restoration of tissue homeostasis and resolution of acute inflammation [5,6].

Neuroinflammation is an inflammatory reaction that takes place in the central nervous system (CNS). However, neuroinflammation is a global process that often encompasses the CNS and involves peripheral responses with cellular players, either resident in the brain or traveling from the periphery, or even acting from the periphery. Many of these cells interact either locally or from a distance through signaling molecules and nerve wire connections [7,8].

The CNS-infiltrating cells in a neuroinflammatory disease such as multiple sclerosis (MS) are mainly composed of encephalitogenic T cells, B cells, and activated macrophage/microglial [9,10]. Various subsets of CD4+ T cells have an important role in MS immunopathogenesis, but T helper T_H_1 and T_H_17 cells have a crucial role in inflammatory development and the effector response [11]. In this context, the presence of dendritic cells (DCs) within the CNS has been suggested for years, but their derivation and their functions during neuroinflammation are not clear yet. Under steady-state conditions, DCs present in CNS act as sentinels, continually sampling their local environment [12,13,14]. They share this function with macrophages derived from the same basic hematopoietic (bone marrow-derived) precursors and with parenchymal microglia that arise from a unique non-hematopoietic origin. While multiple cells may serve as antigen-presenting cells (APCs), DCs present both foreign and self-proteins to naïve T cells that, in turn, carry out effector, helper, and cytotoxic functions that serve as a defense against foreign insults. Thus, DCs are the professional antigen-presenting cells that patrol all the tissues, recognize foreign antigens and present them to T cells to either mount an immune response or induce tolerance. Therefore, it is likely that studying one cell type will only provide a partial view of the whole process. Keeping this in mind, we set this work to revise some of the current knowledge on the participation of DCs in neuroinflammatory conditions, with a special focus on multiple sclerosis: indeed, DCs are reported to be involved in various neuroinflammatory and neurodegenerative disorders, but their actual role is less known than that of other immune cells [15,16]. Given the recent evidence that inhibition of sirtuin 6 hampers DC migration and given the importance of DC migration to the lymph nodes to initiate immune responses, we will also review the role of sirtuins in DCs’ functions and neuroinflammation.

## 2. Dendritic Cells Classification

DCs are bone marrow-derived cells playing a major role in the activation of the immune system and immunosurveillance for their ability to sample the environment, detect the presence of antigens and induce T cell activation. A very old classification of DCs was focused on their anatomical localization: the family of “migratory tissue DC” included all the cells out of lymphoid organs, while “lymphoid resident DC” was restricted to all the cells present in lymphoid organs. In this context, most DCs belong to the “conventional or classical” type and are called cDCs. The other DCs, a rare subset, are called plasmacytoid DCs (pDCs) and are present in blood and lymph nodes (LNs) [17,18]. Nowadays, DC subsets have been classified in mice based on the presence of cell surface markers, i.e., defined by the expression of CD11c and MHC-II, in combination with CD4, CD8α, CD11b, and CD205 [19,20,21]. Of note, CD8α is not expressed by human cDCs, while the expression of CD11c, CD141, and CD16 are used to segregate three human cDC subsets [17,18,22,23]. Three main conventional subsets of CD11c^+^ MHC-II^+^ DCs are identified in physiological condition, including CD4^−^CD8α^+^CD205^+^CD11b^−^, CD4^+^CD8α^−^CD205^−^CD11b^+^, and CD4^−^CD8α^−^CD205^−^CD11b^+^ [24,25]. CD4^−^CD8α^high^CD205^+^CD11b^−^ lymphoid DCs are predominantly present in the thymus, populate the T-cell area of the spleen, and are also found in LNs [26]. It has already been shown that this subset may activate both CD4^+^ and CD8^+^ T cells and concomitantly has a regulatory effect on T cells, eliciting both apoptosis in CD4^+^ T cells and a limited CD8^+^ T cell activation, resulting in the induction of cross-tolerance. In contrast, CD4^−^CD8α^high^CD205^+^CD11b^−^ lymphoid DCs can not only activate CD8^+^ T cells but also cross-present for the stimulation of cytotoxic T cells [27,28].

The CD11b-bearing DCs are thought to be involved in the antigen presentation via MHC class II-restricted antigens to T helper (T_H_), to induce polarization toward T_H_17 and T_H_2 subsets [27]. The CD8a expression on cDCs delineates a specific subset involved in the activation of the cytotoxic response (mediated by CD8 T cells) and in the production of the T_H_1-polarizing cytokine IL-12 [29].

CD4^+^CD8α^−^CD205^−^CD11b^+^ and CD4^−^CD8α^−^CD205^−^CD11b^+^ subsets are located in the marginal zone between white and red pulp [28], and migrate to the T-cell area upon stimulation [30]. The absence of CD8α expression identifies a subset considered as an efficient stimulator of CD4^+^ and CD8^+^ T cells in in vitro settings and can direct a T_H_2-type immune response in vivo. In addition, it can trigger the development of T_H_1 cells in vivo, consistent with activated CD4^−^CD8α^high^CD205^+^CD11b^−^ lymphoid DCs being the major producers of IL-12. Normally found in the spleen as well as in other secondary lymphoid organs and the lymphatic system, two other additional DC subsets have been characterized. They represent the mature form of interstitial tissue DCs which are CD4^−^CD8α^−^CD205^+^CD11b^+^ myeloid and CD4^−^CD8α^low^CD205^high^CD11b^+^ myeloid subsets. They play a fundamental role as efficient activators of CD4^+^ T cells to possibly generate T_H_1 cells [31,32]. More recently, with the increased availability of multi-data analysis, comparative gene expression analyses have highlighted differences in DCs resulting in a new classification based on the differential expression of key transcription factors, such as interferon regulatory factors 8 and 4 (IRF8 and IRF4). Accordingly, the new classification of DCs could be defined by exploring their surface markers and/or transcription factors (Table 1) [33].

pDCs are interferon (IFN)-producing cells, abundant in blood as immature cells, defined by their plasma-cell-like morphology spherical shape, and pDCs play a crucial role in antiviral immunity [34,35,36,37]. A plethora of reports indicates that pDCs are classified as DCs because they can upregulate the expression of MHC-II, CD80, and CD86 upon pro-inflammatory maturation and can induce T cell polarization [38]. Upon activation, pDCs produce and release large amounts of IFN-α and induce CD123 translocation to the plasma membrane, where it is involved in cell growth, proliferation, survival, and differentiation [39].

## 3. Dendritic Cells Shape the Autoimmune Response in Neuroinflammation

As mentioned, DCs are highly specialized antigen-presenting cells (APC) with a crucial role in immune system activation, regulation of immunological tolerance, and autoimmune disorders. From a physiological point of view, DCs are immune cells that link the innate to the adaptive immune systems, with a key role in activating, shaping, and preventing CNS immune-mediated damage that is characteristic of multiple sclerosis (MS) and Experimental Autoimmune Encephalomyelitis (EAE), one of the animal models of MS [40,41]. In the context of autoimmune disorders, such as MS, one of the strongest genetic associations is a polymorphism of the human leukocyte antigen complex (HLA-DRB1*1501), which is closely related to antigen presentation of APC [42], such as DCs; several other HLA region variants are also strongly associated with MS [43]. While the triggering factors in MS are still debated, the key role of T and B cells, activated by the pro-inflammatory form (immunogenic) of DCs, is well established [44] and, in particular, the encephalitogenic triggering of T-cell activation is mediated by professional DCs. In addition, DCs may, in turn, activate the immune response, regulate immunological tolerance (tolerogenic DCs, tolDCs), and regulate the maintenance of CNS immune surveillance [45]. Although their number in physiological conditions is limited, it increases in MS and Myelin Oligodendrocyte Glycoprotein MOG35-55-immunized EAE, suggesting a possible role in neuroinflammation and neurodegeneration [46]. The fundamental role played by DCs is further supported by the fact that many therapies, verified in EAE and approved for humans, lead to significant modifications of DC activation [47]. EAE recapitulates many aspects of the human disease, such as the DC-mediated CNS inflammation, migration of DCs to the LNs, and encephalitogenic T cell and B cell activation, resulting in an attack on oligodendrocytes and demyelination. Although not perfect, EAE has allowed: (i) uncovering some of the molecular pathways governing the trigger factor(s) of MS; (ii) elucidating the pathogenic central role of DCs; (iii) understanding that the loss of immunological tolerance is one of the main pathological mechanisms that lead to the autoimmune response toward the CNS [41,48,49]. Experiments using MOG-immunized mice have also clarified that DCs, in the guise of the different subsets, are present in the periphery and several other subsets of bone marrow-derived MHC-II^+^ cells normally populating the CNS, where they play a fundamental role as demyelinating lesion-initiating DCs [12,50]. Considering the mode of action of DCs, it has already been shown that CD103^+^ cDC1 Langerhan cells, dermal specialized DCs, encounter the relevant myelin antigen and migrate to the LNs where they present it to T cells. Accordingly, DC-mediated presentation of self-neuroantigens is sufficient to induce EAE in naïve mice [51]. However, the identity of the specific DC subset that plays a role in the encephalitogenic activation of T cells in LNs has been challenged when it was found that Batf3-deficient mice, which lack CD103, were susceptible to chronic EAE induced by MOG injection [52]. DCs are found not only in non-lymphoid peripheral tissues but also in most primary lymphoid organs, where Follicular DCs (FDCs) are a specific tissue and specialized type of APC, with a stromal origin [53]. FDCs are largely restricted to lymphoid follicles, in the central region of primary follicles, and in the light zone of germinal centers where they form and maintain the follicular architecture [54,55]. FDCs play a fundamental role in capturing and retaining the native antigen by linking to complementary and immune complexes and then presenting these antigens to germinal center B cells that start the secondary immune response [56,57]. In the context of EAE and MS, naïve B cells migrate to LNs, attracted by the chemokine CXCL13 produced by FDCs, and in LNs the B cells meet the antigen, become activated, and participate in the formation of B cell follicles with germinal centers, where they differentiate into antibody-producing plasma cells [58,59,60]. Conversely, DCs that migrate in the lymphoid organs can originate both from precursor cells, continuously replenished by a mobile blood-derived pool, present in non-lymphoid peripheral tissues, and from precursor cells originating in bone marrow [61,62,63]. When the precursors of DCs encounter the antigen in the periphery, they migrate towards the afferent lymphatic vessel and then in the draining lymph nodes, into the T-cell-rich center. In physiological conditions, there is a continuous low level of traffic from the periphery to LNs; however, in EAE-affected mice, the migration of DCs is greatly increased after subcutaneous injection of myelin-relevant peptides in the peripheral tissue. In LNs of EAE-affected mice, DCs play a key role in initiating T-cell responses [64,65,66,67]. Further characterization of DCs throughout the course of EAE has indicated that different subsets of DCs serve distinct functions: cDCs are involved in disease development, whereas pDCs, which produce interferons, are important in the development of T-regulatory cells and disease resolution [68,69,70].

Different protocols of EAE induction are available: EAE can be induced in susceptible rodent strains by active immunization with myelin antigens, as well as it can be induced by adoptive transfer antigen-specific myelin-reactive T cells. Studies exploiting these EAE models demonstrated that auto-aggressive encephalitogenic T cells migrate into the CNS, upon DC activation, where they recognize their cognate antigen and initiate the neuroinflammatory response leading to axonal damage and neuronal cell death. Although the molecular requirements for naïve T-cell priming and the formation of an immunologic synapse between a naïve T cell and a competent antigen-specific DC are well characterized, little is known regarding peripheral DCs or CNS-resident DCs’ role in the etiopathogenesis of MS and EAE. Moreover, the potential role of DCs in MS and EAE pathogenesis is complicated by the fact that DCs are heterogeneous, with a range of functional phenotypes, being pro-inflammatory or tolerogenic under certain conditions [71]. Different subsets of bone marrow-derived MHC-II^+^ cells’ professional DCs are present within the CNS in the cerebrospinal fluid, choroid plexuses, meninges, and perivascular spaces, in both mice and humans [50,72]. In addition, MHC-II^+^ cells with characteristics typical of DCs, i.e., based on cell surface marker expression, morphology, and/or ultrastructural characteristics, are normal constituents of the choroid plexus, meninges, and perivascular spaces in the uninjured CNS of both humans and rodents [14,45,72,73]. However, the rare DCs that populate CNS in steady-state conditions, might be recruited to these sites as preDCs and only differentiate into APC in the brain parenchyma [74].

A major role of CNS-resident DCs during neuroinflammation is to reinforce the antigen presentation to T cells to license them to cross the blood-brain barrier and invade the CNS parenchyma [75]. Specifically, CNS-resident DCs drive the polarization of T cells toward a T_H_17 phenotype resulting in the secretion of IL-17 and GM-CSF to maintain their pathogenic properties. In pathological conditions, the CNS-resident DCs not only support the reactivation of antigen-experienced T cells but also promote the priming of naïve T cells, which has been considered a potential mechanism of epitope spreading [67]. These CNS-resident DCs are placed in ideal sites where they interact with infiltrating T cells, since the choroid plexus and meninges, as well as CNS parenchymal blood vessels, are important portals for leukocyte entry during EAE and MS [76,77]. It has already been demonstrated, using transgenic mice in which the expression of MHC-II is restricted to CD11c^+^, a DCs specific marker, that DCs are susceptible to EAE, suggesting that DCs alone are sufficient to activate the T cells’ encephalitogenic response in vivo and, thereby, promote their local polarization in effector cells. Hence, it is clear that DCs can activate self-antigen-specific naïve T cells in peripheral LNs and subsequently promote their differentiation into effector T_H_1 and T_H_17 cells: the interplay between DCs and encephalitogenic T cells is crucial in triggering EAE and MS.

DCs are thought to be natural modulators because they are involved in the polarization of naive T cells, providing all necessary signals for T cell activation or immunomodulation, thereby defining the outcome of the immune response in health and disease [78,79]. In the context of neuroinflammatory disorders, Yonghao Cao and coworkers identified a comparable number of myelin-reactive T cells in MS patients and healthy controls. In contrast to healthy controls, libraries derived from myelin-reactive T cells of MS patients exhibited significantly enhanced production of pro-inflammatory cytokines and reduced production of the anti-inflammatory cytokine IL-10. Altogether these data demonstrated that antigen-specific T cells from MS patients functionally differ from healthy controls [80] and thus, immunogenic activation of DCs in the periphery is a crucial step of the autoimmune response mediated by the myelin-reactive T cells [81,82]. In addition, activated myelin-reactive T cells are then reactivated after the engagement of CNS-resident DCs, which present myelin-derived epitopes or reinforce T cell activation [83]. However, activation of T helper cells and cytotoxic T cells differs between humans and mice. In fact, EAE models are mainly based on auto-reactive T helper cells while in contrast, in MS, cytotoxic T cells and B cells are the main pathological player propagating inflammation and determining demyelination [41,84,85].

Moreover, DCs also promote immune homeostasis by establishing and maintaining peripheral T cell tolerance. In contrast to the naturally occurring regulatory T cells (T_reg_), DCs play an eminent role in the generation of induced T_reg_ in the periphery, where DCs have been reported to control their development and maintenance by playing the immunomodulatory role [86].

## 4. Immunomodulatory DCs in the Context of Neuroinflammation

While the triggering factors in MS are still debated, the key role of the immune system and particularly the autoimmune attack toward myelin is well established [44,87]. Although the number of circulating CNS-reactive T cells present in MS patients and healthy subjects is similar [88], T cells are a key mediator of disease activity in MS [87] and EAE [89]. Thus, the presence of CNS antigen-reactive T cells alone is not sufficient to induce the disease and additional factors are involved. Since antigen presentation, most commonly by DCs, is essential for most T-cell responses [90], it is reasonable to assume that regulation by DCs will provide important checkpoints and balances for the T cell autoimmune response, suggesting that DCs are key players in the immune-pathogenesis of MS and EAE. DCs drive the immune system activation and play a fundamental role in inducing antigen-specific immunity by presenting antigens to naïve T cells and differentiating the antigen-specific T cells into effector T cells. On the other hand, DCs promote and drive self-tolerance through their ability to present self-antigens to developing T cells directly in the thymus in a biological process called central tolerance (negative selection). The mechanism of negative selection in the thymus selects autoreactive thymocytes, induces their apoptosis, and promotes T cell polarization into T_reg_ cells [91]; and this role is mainly ascribed to thymic DCs [92,93]. Specialized DCs play a role in eliminating autoreactive thymocytes, through the expression of MHC-II (IE) that is sufficient to induce apoptosis, via negative but not positive selection, in thymocytes specific for self-antigens [94]. In autoimmune disorders, central tolerance mediated by negative and positive selection is defective and incomplete, and self-reactive T and B cells might drive pathological processes. To counteract this aberrant and potentially pathological mechanism, peripheral tolerance and immunomodulatory biological processes have been evolved to limit the autoimmune reaction. In this context, recent advances in peripheral tolDC biology have revealed their tolerogenic role and have shown their immense therapeutic potential for treating a variety of immune disorders [95], and this potential relies on their capacity to establish an intricate network of cell-to-cell interactions with immune cells, via direct contact and the release of soluble factors in the extracellular milieu. tolDCs expressing Immunoglobulin-like transcript 2 (ILT2), ILT3, and ILT4 inhibitory receptors can play a critical role in immunomodulation and tolerance by promoting inducible-Foxp3^+^ regulatory T cells (iT_reg_) induction. iT_reg_ are generated in the periphery by tolDCs and their generation appears to be dependent on indoleamine 2,3-dioxygenase (IDO), retinoic acid, Vitamin D3, and TGF-β [96]. Moreover, tolDCs secrete higher levels of IL-10 and IL-4 and drive differentiation of naïve CD4^+^ T cells into IL-10 secreting T_reg_ in mice [97] and humans [98].

On the other hand, antigen presentation by DCs without inappropriate co-stimulatory molecule expression (tolDC) leads to T cell anergy or induction of the T-regulatory phenotype [99]. Several stimulations of peripheral CD4^+^ T cells by tolDC can also drive T_reg_ generation, and T cells cultured under stimulation by tolDC selectively upregulate the anti-inflammatory cytotoxic T-lymphocyte antigen 4 (CTLA4) and lose their ability to produce IFN-γ and IL-2 and subsequently differentiate into T_reg_ cells [100]. The secretion of extracellular vesicles (EVs) emerged as another important mechanism used by DCs to deliver complex messages in the microenvironment [101]. EVs are nanosized membranous structures made of a lipid bilayer, virtually secreted by all cell types [102], playing several roles in the biology of the cell: sometimes, they are used as a “waste system”, other times they are exploited as “postmen” to deliver information toward target cells [103]. In particular, DCs release EVs with different cargoes depending on their maturation or activation [104]. EVs from mature DCs were shown to induce an immune response by spreading MHC-antigen complexes to other DCs and also to both CD4^+^ and CD8^+^ T cells, thus mediating their activation [65]. In other disease contexts, EVs secreted by tolDCs can induce tolerance in different animal models of transplantation [105].

All the tolerogenic mechanisms in the DCs armamentarium result in the generation of T_H_2 and T_reg_ from naïve T-cells that represent the effector cells in limiting the extent and magnitude of the encephalitogenic response in MS and EAE. T_H_2 and T_reg_ generate mainly immunomodulatory molecules, such as anti-inflammatory cytokines and surface receptors commensurate with an anti-inflammatory phenotype [105,106,107,108,109,110,111,112,113]. Accordingly, it is fairish to elaborate that DCs play a fundamental role in neuroprotection when their biological asset is commensurate with tolerogenic phenotype. Indeed, Hongmei Li and co-workers demonstrated that intravenous injection of MOG35-55 in EAE induces immune tolerance by an increase in tolerogenic CD11c^+^CD11b^+^ DCs in CNS. The increased number of those DCs results in antigen-specific T_H_2 and T_reg_, which are involved in the induction of systemic tolerance and less demyelination in CNS [114]. Moreover, the tolDCs suppress the development of EAE both directly in the host and therapeutically when infused into recipient EAE-affected mice. Another study revealed that an agonist of aryl hydrocarbon receptor loaded in nanoliposomes together with a T cell epitope from MOG35-55, induced tolDCs and suppressed the disease course of chronic-progressive EAE, and this was associated with the generation of MOG35-55-specific T_reg_, concomitant with less neurodegeneration [115].

Overexpression of the Hepatocyte Growth Factor in neurons of transgenic mice inhibits the development of EAE. The mode of action is mediated by the inhibition of immunogenic DCs that results in the generation of IL-10-producing T_reg_ cells, and down-regulation of surface markers of T-cell encephalitogenic activation. In this study, a histological evaluation revealed that there was less inflammatory infiltrate and less demyelinated area indicating that the reduced activation of DCs could drive neuroprotection [116]. Importantly, employing a transgenic mice model, Roy Y Kim and collaborators demonstrated that targeting Estrogen receptor beta (ERβ) signaling pathways in DCs induces a tolerogenic and immunomodulatory phenotype. Of note, ligands of ERβ enhance remyelination through a direct effect on oligodendrocytes, demonstrating that DCs could mediate the maturation of oligodendrocytes [117].

In the context of MS, several disease-modifying drugs are now being used in therapy but they are neither specific nor selective for MS, as they act as general immunomodulatory agents. Moreover, treatment-related side effects or risks can be severe, leaving a significant and unmet need for safer and more disease-selective treatments. To overcome these problems, the use of tolDCs has been considered. In the following section, we will discuss how the current drugs for MS may impact the immunogenic role of DCs and at the same time, induce tolDCs with the final goal of inducing neuroprotection.

## 5. Disease-Modifying Drugs (DMDs) Targeting DCs in MS

It is now well-accepted that DCs, in their immunogenic activation, also contribute to the pathogenesis of MS. In MS patients, DCs are present in brain lesions and meninges and display an altered phenotype associated with pro-inflammatory function as compared with DCs of healthy controls. However, none of the approved therapeutic drugs or monoclonal antibodies for MS specifically target DCs. However, DMD therapies, at least in part, affect innate immune functions, such as DC-mediated polarization of effector T cells or DC-mediated expansion of regulatory cells, or cell infiltration into CNS inflammation sites of effector cells.

The main therapies to treat MS include immunomodulators [47,118,119,120,121,122,123,124,125,126,127,128,129], anti-α4-integrin (anti-VLA-4) [130,131,132], anti-CD20 antibodies [133], anti-CD52 monoclonal antibodies [134], and selective sphingosine-1-phosphate receptors [135,136,137].

The first class of drugs approved for MS, Interferons, reduces monocytes activation and prevents the pro-inflammatory profile of DCs through a decreased IL-12 secretion. The inhibition of IL-12 production by immunogenic DCs, mediated by type I interferons, results in reduced polarization of T cells toward a pro-inflammatory T_H_1 profile [138].

Natalizumab is a humanized monoclonal antibody that selectively binds to very late antigen-4 (VLA-4 or α4β1-integrin) and it is approved for use in relapsing-remitting MS patients. Natalizumab reduces the encephalitogenic leukocyte homing into the CNS by blocking the molecular interaction between α4β1 expressed by immune cells and Vascular Cell Adhesion Molecule-1, expressed by vascular endothelial cells [139]. Regarding DCs, natalizumab mediates the production of tolerogenic markers in DCs, such as HLA-G and CD274, which may contribute to the reduction of neurodegeneration in MS patients [140]. In addition, the effect of natalizumab may drive the inhibition of DC infiltration in the CNS, but may also impair the DC-mediated T cell activation [132]. Another study revealed that prolonged therapy with natalizumab reduces the number of DCs and the expression of MHCs in cerebral perivascular spaces, suggesting a new possible role for VLA-4 in the infiltration of these cells into the CNS [131].

Fingolimod, a modulator of sphingosine-1-phosphate (S1P) receptors, controls lymphocyte trafficking [141]. Additionally, it acts as an immunomodulator by decreasing the pro-inflammatory activation of DCs, dampening DC-dependent T cell encephalitogenic activation [142,143,144].

Despite the increased efficacy of these treatments, a few pieces of evidence indicate life-threatening side effects that may limit their use in the clinic, as a consequence of the general immunosuppression of these drugs, which impairs the protective and physiological immune system functions. For instance, natalizumab therapy was demonstrated to be associated with an increased risk of progressive multifocal leukoencephalopathy, caused by the JC virus [145].

Another therapeutical strategy, explored in several clinical trials, is the administration of the antigenic peptide, or a mixture of different peptides, to trigger hypo-sensitization and immunological alterations, such as a cytokine shift from the autoimmune T_H_1/T_H_17 profile to the induction of IL-10-secreting T_reg_ [146,147,148,149].

## 6. Alternative Strategies to Target DCs in Autoimmune Disorders

There are several therapies used for autoimmune diseases, such as rheumatoid arthritis (RA), that specifically target DCs. These therapies are not formally approved for MS, but some of them proved to be promising in different clinical trials.

The therapies that target DCs could inhibit immunogenic DCs functions or promote their tolerogenic generation.

Therapies that inhibit DCs maturation toward an immunogenic phenotype include those targeting cytokines production, such as anakinra (recombinant IL-1Ra), tocilizumab [150], MOR103 [151,152], KB003, and BVDU [153], and those targeting the co-stimulating molecules that provide positive signals to T cell activation, such as CTLA 4-Ig. In addition, there are several recombinant chimeric antibodies, such as anti-DEC205-MOG [154] and anti-CD11c-MOG [155], targeting DCs’ receptors and delivering pro-tolerogenic antigens to DCs. Since these recombinant chimeric antibodies are developed using various specific DCs’ receptors as a target, each one can produce dissimilar reactions on different DCs subtypes [156,157,158,159,160,161]. Another promising treatment for MS that directly involves DCs is the ex vivo tolDC therapy [162]. Unfortunately, this approach remains challenging due to the difficulties in the isolation, purification, and culture of autologous primary DCs (or more commonly, their progenitor cells) that require extensive handling and can be prohibitively expensive [163]. An emerging alternative that overcomes both of these limitations is the administration of nanoparticles containing antigens and immunomodulators which can induce tolDCs [164]. This approach benefits from antibodies covering the nanoparticle to target specific DCs’ receptors, such as DEC205 or Clec9A, thus delivering specifically to these cells the content the particles were loaded with, i.e., immunomodulators, such as rapamycin [165], myelin antigen [166], IL-10, and combinations of autoantigen and immunomodulator [167,168,169,170].

Finally, preventing the migration of DCs may represent an alternative strategy. Indeed, DCs transport antigens from sites of inflammation to the lymph organs for immune activation. A neuronal plasticity molecule-activity-regulated cytoskeleton-associated protein/activity-regulated gene 3.1 (Arc/Arg3.1), expressed in migratory dendritic cells in the skin, was identified [171]. Arc/Arg3.1 regulates cytoskeletal changes in DCs, accelerating migration in response to inflammation. Moreover, Arc/Arg3.1 is required for inducing T cell responses in two different disease models—EAE and allergic contact dermatitis. Targeting Arc/Arg3.1 may therefore be exploited to selectively modify immune responses [171]. Inhibition of the NAD^+^-dependent deac(et)ylase sirtuin 6 was demonstrated to delay the onset of Experimental Autoimmune Encephalomyelitis, by dampening DC migration towards inflamed LNs (see below).

## 7. Role of Sirtuins in DCs

Given our recent evidence that inhibition of sirtuin 6 delays EAE onset by dampening DC trafficking towards inflamed LNs, we will discuss in the following paragraphs the role of sirtuins in DCs’ functions and in neuroinflammation.

Sirtuins are NAD^+^-dependent deac(et)ylases involved in the control of important pathways in different cell types and under different stimuli. The sirtuins’ family is composed of seven members (named from SIRT1 to 7) which differ in cellular localization, substrate specificity, and physio-pathological role inside the cells. SIRT1, 6, and 7 have a nuclear localization and their role is to deac(et)ylate histones (with different lysine specificity on histone 3) and other proteins (such as p53, MYC, FOXO, NF-κB, HIF-1α, etc.) on lysine residues to control gene expression. SIRT3, 4, and 5 are localized in the mitochondria. The major role of SIRT3 is to control energy metabolism in the mitochondria, including fatty acid oxidation, and maintain basal ATP levels regulating the electron transport chain through deacetylation of several factors [172]. SIRT4 seems to exert the opposite effect in respect of SIRT3 and SIRT5. Indeed, SIRT4 negatively regulates glutamine catabolism, fatty acid oxidation, and amino acid catabolism [172]. SIRT5 has a predominant demalonylation, desuccinylation, and deglutarylation activity controlling glycolysis and gluconeogenesis [172]. Finally, SIRT2 is predominantly localized in the cytosol and its primary activity is to control cell cycle, genomic integrity, microtubule dynamics, cell differentiation, metabolic networks, and autophagy through the deacetylation of several proteins among which α-tubulin, CDC20, H4K16.

The sirtuins’ world is complex: the literature on sirtuins is exponentially growing and the presented results are sometimes conflicting. Monocytes/macrophages and microglia, together with DCs, form the system of mononuclear phagocytes. The role of sirtuins in microglia and neuroinflammation is discussed in paragraph 8. In monocytes/macrophages, the role of sirtuins has been investigated in many different contexts and diseases. The complete discussion on the regulation of different processes in these cells by sirtuins is out of the scope of this review, and we mention just a few reports: (a) SIRT1 inhibits monocyte to macrophage differentiation [173] and, in the context of atherosclerosis, SIRT1 deficiency in monocytes/macrophages contributes to increased oxidative stress, inflammation, foam cell formation, senescence impaired nitric oxide production and autophagy, suggesting that SIRT1 activation may be a new therapeutic strategy against atherosclerosis [174]; (b) SIRT3 protects against asbestos-induced pulmonary fibrosis by mitigating monocyte-derived alveolar macrophages recruitment [175], suppresses renal calcium oxalate crystals by promoting macrophages M2 polarization [176], and the absence of SIRT3 impairs autophagy in macrophages [177]; (c) SIRT6 inhibition was reported to suppress macrophage migration, phagocytosis, and M2 polarization [178], whereas in the context of diabetic nephropathy, SIRT6 overexpression determined macrophage M2 polarization [179] and, in the context of rheumatoid arthritis, SIRT6 deficiency increased macrophage-mediated inflammatory processes [180].

In this paragraph, we will focus on the role of sirtuins in DCs, in which the most studied sirtuins are SIRT1 and SIRT6. The effects of sirtuins on DCs are summarized in Table 2.

### 7.1. Sirtuin 1

SIRT1 seems to have a role in the maturation of CD4^+^ T_reg_ lineage and the repression of T_H_1 [181]. Naïve CD4^+^ T cells, incubated with DCs expressing low levels of SIRT1, presented an increased expression of IFN-γ but not of IL-17A or IL-4. This observation reveals a differentiation of naïve CD4^+^ cells in the T_H_1 lineage [181]. In addition, when SIRT1 expression is downregulated, DCs exhibit an increased level of phosphorylated STAT4 and a decreased level of phosphorylated SMAD3, which bring about an enhanced expression of IL-12 and a reduced expression of TGF-β [181]. These two cytokines are responsible for the alteration of T_H_1 and T_reg_ differentiation through the increased expression of IL-12 receptor and decreased expression of TFG-β receptor in naïve CD4^+^ T cells [181]. On the contrary, Woo and colleagues showed, with a knockout mouse model for SIRT1, that SIRT1 is involved in the maturation of bone marrow CD80^+^ CD86^+^ DCs and, consequently, in the maturation of T_H_1 and T_H_17 cells from naïve CD4^+^ [182]. In addition, DCs derived from patients affected by rheumatoid arthritis (RA) possess an increased expression of SIRT1 which determines the increased inflammatory response of this autoimmune pathology [182]. Further evidence of the pro-inflammatory role of SIRT1 in DCs derives from another study on airway allergy, Legutko and colleagues showed that SIRT1 is responsible for T_H_2 cells activation by DCs via PPAR-γ repression [112]. From these data, the pharmacological modulation of SIRT1 could be used to drive the immunity response in a DC-type-related manner. In other words, the inhibition or activation of SIRT1 should be chosen related to the specific condition to direct the maturation of the CD4^+^ lineage most suitable for facing the specific inflammatory state.

### 7.2. Sirtuin 6

Among the different sirtuins, SIRT6 role in DCs differentiation and functions has been deeply investigated, also in the context of EAE, an animal model to study MS (see below for SIRT6 inhibition as an approach in EAE).

SIRT6 role in the regulation of processes is being increasingly recognized, and it is covering many different functions, including energy metabolism (related to both glucose and lipids), DNA repair, aging, inflammation, and immunity. In these latter respects, it can be mentioned that SIRT6 regulates tumor necrosis factor α (TNF-α) release, through two different mechanisms: at a post-translational level, SIRT6 deacylase activity removes fatty acyl groups from target lysines in TNF-α [185]; at a transcriptional level, TNF-α release is regulated by SIRT6, through a TRPM2-mediated Ca^2+^-dependent mechanism [186]. Nevertheless, regarding immune cells, we will specifically focus on the SIRT6 role in the regulation of DC functions, extensively investigated by Dr. Lasigliè and colleagues [183].

SIRT6 promotes myeloid conventional DC (cDCs), but not plasmacytoid DC, differentiation, and maturation (both spontaneous and induced by TLR ligand) and function. This conclusion was obtained with different approaches, also using SIRT6 KO mice, and pointing to a specific role for SIRT6 in certain DCs lineages. Bone marrow (BM)-derived cDCs (BMDCs) were not properly generated in the absence of SIRT6, and this result may be ascribed to the impaired generation of TNF-α, essential for BMDC differentiation and maturation [187,188,189]. The expression of maturation markers (MHC-II, CD80, CD86, and CD40) was strongly reduced in BMDCs from SIRT6 KO compared to those from wild-type mice. SIRT6 is also important in the regulation of cytokine release from DCs: in basal conditions, SIRT6 depletion determined the reduced expression of TNF-α (as already demonstrated in other immune cells, see above), and IL-12; conversely, IL-6 release was increased in the absence of SIRT6. In stimulated conditions, TNF-α was increased in SIRT6 KO DCs.

Overall, this study revealed that SIRT6 is crucial in DC biology.

## 8. Role of Sirtuins in Neuroinflammation

The role of sirtuins in neuroinflammation is controversial. Some studies documented a protective role of sirtuins, whereas other studies reported that sirtuins have pro-inflammatory effects. In particular, the most studied sirtuin in neuroinflammation seems to be SIRT1. In addition, also the role of SIRT6 and SIRT7 has been explored.

### 8.1. Sirtuin 1

SIRT1 has been shown to have a protective effect on neuroinflammation by the deacetylation, and consequent inactivation, of NF-κB p65 [190]. The inactivation of NF-κB brings about the suppression of microglial activation and neuroinflammation, as reported in several studies [191,192,193,194]. In addition, Chen and colleagues showed that the activation of SIRT1 is a neuroinflammation protective factor in spinal cord injury, reducing the number of macrophages/microglia in the area of damage and promoting locomotor recovery [195]. In support of the role of SIRT1 as a modulator of inflammation, another report demonstrated that SIRT1 downregulation, controlled by miR-132, increases the production of pro-inflammatory cytokines such as TNF-α and lymphotoxin in B cells [196].

Several studies investigated the role of SIRT1 in the inflammatory demyelinating disease MS. EAE is the most commonly used experimental model for human MS. In vivo experiments using this animal model showed that the conditional overexpression of SIRT1 in the brain decreases the clinical severity of EAE by reducing T cell infiltration within the spinal cord parenchyma, decreasing pro-inflammatory cytokines (IFN-γ and IL-17), but increasing the anti-inflammatory cytokine IL-10 [197]. In addition, the expression of SIRT1 is decreased in PBMCs of relapsing patients [198]. On the contrary, SIRT1 expression is elevated in oligodendrocytes and GFAP-positive astrocytes in acute and chronic MS lesions [199], and SIRT1 acts as a cell cycle arresting factor in differentiating oligodendrocyte progenitor cells [200,201,202]. Further in vivo experiments demonstrated that SIRT1 can stimulate autoimmune responses by activating T_H_17 cells, and the pharmacological treatment with SIRT1 inhibitors ameliorated the phenotype of EAE mice, whereas treatment with resveratrol (a SIRT1 activator) significantly exacerbated demyelination and inflammation [203,204].

There are several pieces of evidence that SIRT1 signaling activation could ameliorate EAE mouse model phenotype attenuating demyelination, axonal loss, oligodendrocyte apoptosis, oligodendrocyte progenitor cell recruitment, decreasing pro-inflammatory cytokine expression, and enhancing anti-inflammatory cytokine expression [106,190,205,206]. In addition, several studies demonstrated that ketogenic diet, polyphenols, and SIRT1 activators have a neuroprotective role by increasing the expression of SIRT1 in astrocytes, microglia, and mature oligodendrocytes, reducing oxidative stress and modulating the SIRT1/PPAR-γ and SIRT1/P-AKT/mTOR pathways [207,208,209,210,211,212].

Altogether these data show a cell lineage-dependent role of SIRT1 in neuroinflammation, suggesting that the possibility of pharmacologically targeting SIRT1 to decrease myelin degeneration in MS patients should be developed by a cell-specific delivery of SIRT1 modulating drugs.

### 8.2. Sirtuin 2

Regarding SIRT2, in line with the general trend of contrasting results in the sirtuins’ world, even regarding its role in microglia and neuroinflammation studies are reporting conflicting results. On one hand, Pais and colleagues showed a decrease in microglia activation and neuroinflammation in a process dependent on serine 331 (S331) phosphorylation [213]. When SIRT2 is phosphorylated in serine 331 its deacetylase activity is inhibited, thus it can not deacetylate NF-κB to block the expression of inflammatory cytokines [213]. In contrast, Wang and colleagues showed that the inhibition of SIRT2 by its inhibitor AGK2 brings about a significant decrease in LPS-induced expression of neuroinflammation genes in microglia in vivo by blocking the nuclear translocation of NF-κB [214].

### 8.3. Sirtuin 3

The role of SIRT3 in neuroinflammation was investigated only in recent years. Zhou and colleagues showed that SIRT3 has a role in controlling LPS-mediated mitochondrial damage by suppressing mitochondrial fission and blocking mitochondria-mediated apoptosis in microglia cells [215]. Thus the pharmacological activation of SIRT3 was proposed as an effective treatment to prevent neuroinflammation.

### 8.4. Sirtuin 6

Along with the possibility of inhibiting SIRT1, also the pharmacological inhibition of SIRT6 has been proposed as a strategy in EAE. Few commercially available SIRT6 modulators are available, and this hampered the possibility of performing in vivo studies on the pharmacological modulation of SIRT6. A few small molecules inhibiting SIRT6 have been identified through in silico screens [216,217,218]: one compound (named **1**), with quinazolinedione structure, was the first (and only, so far) SIRT6 inhibitor used in in vivo pre-clinical trials. At first, it was used in a model of Type 2 diabetes, and **1** ameliorated different parameters [219]. Next, **1** was used in mice with EAE. The rationale for exploring the possible effect of SIRT6 inhibitors in EAE was based on some premises: (a) SIRT6 promotes the release of TNF-α from different cells (including DC) through different mechanisms [183,185,220,221]; (b) SIRT6 promotes the secretion of IFN-γ and IL-8 [186,221,222]; (c) SIRT6 enhances DC differentiation and maturation [183].

**Figure 1:**
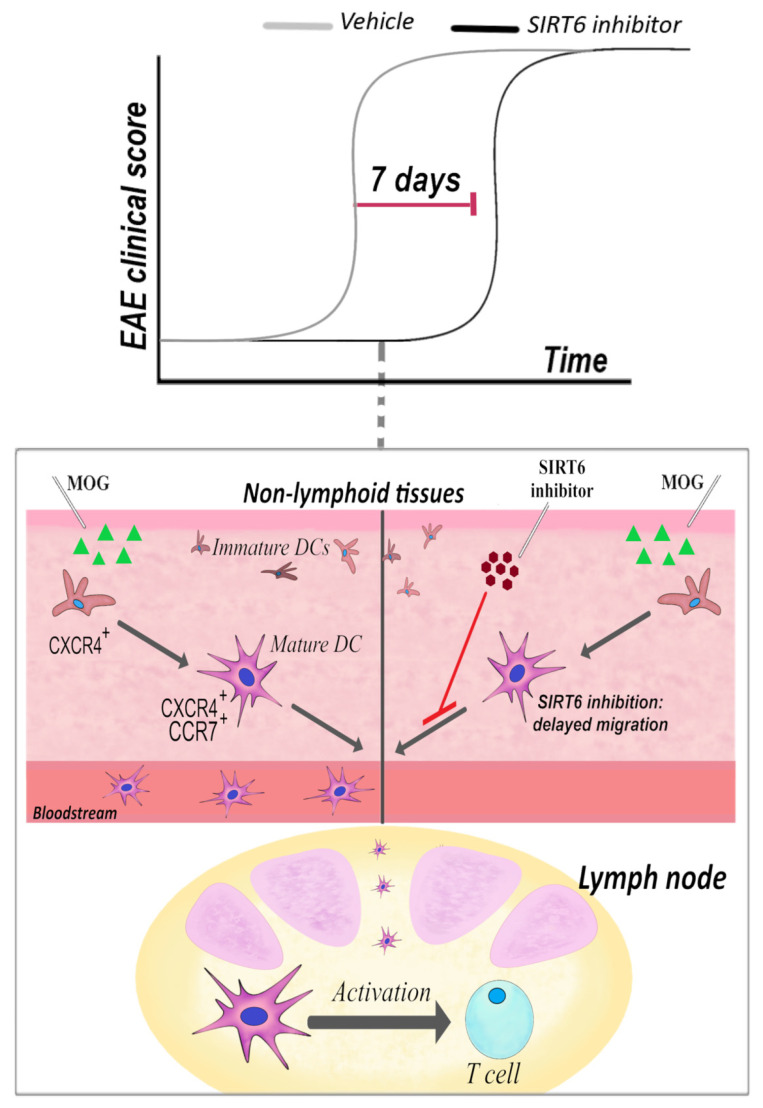
Sirt6 inhibition delays EAE onset by reducing DC migration. In a “preventive” treatment, a SIRT6 inhibitor, named 1, was administered intraperitoneally (30 mg/kg, once/day) at 3 days post-immunization: the clinical score reveals that disease onset was greatly delayed. The representation of CXCR4^+^ and CXCR4^+^/CCR7^+^ DCs in lymph nodes was greatly reduced [184].

The SIRT6 inhibitor, **1**, was administered following both a “preventive” and a “therapeutic” protocol. The therapeutic effect in EAE was rather disappointing. Instead, SIRT6 inhibition strikingly delayed EAE onset, i.e., it had a great impact on the disease onset if administered well before the appearance of clinical symptoms. Figure 1 summarizes the effect of the SIRT6 inhibitor when administrated in the “preventive” protocol. This protocol was aimed at investigating the effect of **1** during the early phase of the disease, at the beginning of the inflammation (triggered with the antigen administration) that then leads to the disease. In line with SIRT6 role in pro-inflammatory responses, SIRT6 inhibition delayed the inflammatory events that follow mice immunization and precede EAE onset. Of relevance for this review, among the other anti-inflammatory effects, SIRT6 inhibition reduced the percentage of CXCR4-positive and CXCR4/CCR7 double-positive DCs in lymph nodes [184], with CXCR4 and CCR7 two pro-migratory surface markers. Therefore, possibly, the delay in the onset of EAE, obtained upon **1** administration, may reflect the reduced DC migration. Indeed, in vitro experiments also confirmed that DC migration was impaired by SIRT6 inhibition [184]. The delayed migration may be a result of the reduced TNF-α production by different cell types [184], which is crucial for DC activation and migration [223,224].

Overall, this study suggests that SIRT6 inhibition may be exploited for the treatment of MS or other autoimmune disorders for its effects on the DCs. Likely, SIRT6 inhibition will not be considered as a possible strategy for overt MS, as it failed to have an impact on the “therapeutic” protocol. Instead, SIRT6 inhibition may deserve further studies for the treatment of patients at early stages, and/or in the “clinically isolated syndrome” (CIS): few options are available to stop progression to MS [225]. Notably, patients with CIS have a high frequency of DCs in their peripheral blood, and SIRT6 inhibition may at least delay the progression to MS by interfering with DC migration.

### 8.5. Sirtuin 7

Regarding SIRT7, Burg and colleagues have demonstrated that the epigenetic factor SIRT7 influences the immune and nervous system, but its role is too weak to be of modulating relevance in the clinical score in experimental autoimmune neuroinflammation [226]. In particular, SIRT7 KO mice did not show differences in disease onset, disease severity, or remission when compared to wild-type mice. However, SIRT7 KO mice showed a slight decrease in the number of T_reg_ cells in the CNS during the chronic phase of EAE [226].

## 9. Conclusions

DCs play a major role in immune system activation and are considered a key factor of MS etiopathogenesis. Accordingly, manipulation of their phenotype could be attractive for treating autoimmune disorders. Indeed, DCs have shown their immense therapeutic potential for treating a variety of immune disorders when they are educated to be tolerogenic. In this context, SIRT6 inhibition may deserve further investigation as a strategy to affect immunogenic DCs.

## Figures and Tables

**Table 1:** Classification of CD11c^+^MHCII^+^ dendritic cells.

Type	Anatomical Sites	Surface Markers				Main Functions
		CD4	CD8α	CD205	CD11b	
Lymphoid	Thymus, spleen, lymph nodes	−	high	+	−	Induction of cross-tolerance, antigen cross preservation for the stimulation of cytotoxic response
		+	−	−	+	Lymphocyte polarization toward T helper
		−	−	−	+	Lymphocyte polarization toward T helper
Myeloid	Spleen, lymph nodes, lymphatic system	−	−	+	+	CD4^+^ T cell activation toward T_H_1 generation
		−	low	high	−	

**Table 2:** Sirtuins effects on dendritic cells and derived effects on immune cells.

Sirtuin	Effects on DCs	Derived Effects on Immune Cells	Ref
Sirtuin 1	↓IL-12; ↑TGF-β	↓T_H_1 and ↑T_reg_ differentiation	Liu G. et al. 2015 [181]
	promotes maturation of CD80^+^CD86^+^ in mice DCs	T_H_1 and T_H_17 differentiation	Woo S.J. et al. 2016 [182]
	↑IL-12p70, IL-1β, and IL-6; ↓IL-10		
	↓PPARγ; ↑IL-4, IL-5, IL-13 in lung DCs	↑T_H_2 maturation	Legutko A. et al. 2011 [112]
Sirtuin 6	↑TNF-α, MHC-II, CD80, CD86, CD40, IL-12; ↓IL-6 in CD11c^+^ BMDCs	-	Lasigliè D. et al. 2016 [183]
	promotes maturation of CD11c^+^ BMDCs		
	↑CXCR4 and CCR7	-	Ferrara G. et al. 2020 [184]
	promotes migration of DCs to lymph nodes		

The effects of each sirtuin on DCs are described as a list of modulated markers and cytokines. The consequent effects that activated DCs have on immune cells are also summarized.

## Data Availability

The data that support this manuscript are publicly available from manuscripts found on PubMed.
